# The Law—Explanatory

**Published:** 1870-07

**Authors:** 


### THE LAW—EXPLANATORY.

We are frequently asked as to the mode of procedure against those who are practicing Dentistry in violation of the law regulating the same; and, in the first place, we will simply quote from the third section of the act:

"All prosecutions under this act shall be by indictment before the Court of Common Pleas, where the offense was committed."

Prosecutions being by indictment, it is proper for any person being aware of a violation of the law, to make known the fact to the Prosecuting Attorney of the county where such offense has been committed; giving at the same time the names of any persons who may be witnesses. A grand jury finds a bill, if the evidence will warrant; the case will then be brought before the court for trial.

We would suggest, however, to our professional brethren throughout the State, that before adopting this course they should notify any violators of the law, that they may have an opportunity to set themselves right, before being subjected to a prosecution. We trust there may never be a prosecution under this law; but that all within our State will conform to its requirements. But for any person to commence the practice of Dentistry in the State of Ohio, at any time since May 8th, 1868, without conforming to the requirements of the law, is certainly doing great injustice to those who have conformed, and this latter class should see to it that none, with impunity, trample upon their rights.

